# Short-form Ron is a novel determinant of ovarian cancer initiation and progression

**DOI:** 10.18632/genesandcancer.109

**Published:** 2016-05

**Authors:** Katherine M. Moxley, Luyao Wang, Alana L. Welm, Magdalena Bieniasz

**Affiliations:** ^1^ Department of Obstetrics and Gynecology, Division of Gynecologic Oncology, University of Oklahoma Health Science Center, Oklahoma City, Oklahoma, USA; ^2^ Functional and Chemical Genomics Program, Oklahoma Medical Research Foundation, Oklahoma City, Oklahoma, USA; ^3^ Department of Oncological Sciences, University of Utah, Salt Lake City, Utah, USA

**Keywords:** sfRon, high-grade serous ovarian cancer, PDK1, ovarian cancer, PI3K

## Abstract

Short-form Ron (sfRon) is an understudied, alternative isoform of the full-length Ron receptor tyrosine kinase. In contrast to Ron, which has been shown to be an important player in many cancers, little is known about the role of sfRon in cancer pathogenesis. Here we report the striking discovery that sfRon expression is required for development of carcinogen-induced malignant ovarian tumors in mice. We also show that sfRon is expressed in several subtypes of human ovarian cancer including high-grade serous carcinomas, which is in contrast to no detectable expression in healthy ovaries. In addition, we report that introduction of sfRon into OVCAR3 cells resulted in epithelial-to-mesenchymal transition, activation of the PI3K and PDK1 pathway, and inhibition of the MAPK pathway. We demonstrated that sfRon confers an aggressive cancer phenotype *in vitro* characterized by increased proliferation and migration, and decreased adhesion of ovarian cancer cells. Moreover, the *in vivo* studies show that OVCAR3 tumors expressing sfRon exhibit significantly more robust growth and spreading to the abdominal cavity when compared with the parental sfRon negative OVCAR3 cells. These data suggest that sfRon plays a significant role in ovarian cancer initiation and progression, and may represent a promising therapeutic target for ovarian cancer treatment.

## INTRODUCTION

There is a great interest in understanding key mediators of carcinogenesis and tumor progression, in order to develop preventive or therapeutic strategies to combat cancer. The receptor tyrosine kinase Ron, which belongs to the MET protooncogene family, has been a focus of cancer research for the last two decades [1]. It has been documented that Ron is involved in the pathogenesis of several malignancies, where its expression usually correlates with more aggressive disease and poor cancer specific outcomes [2]. Often concomitant with the expression of Ron is the expression of an alternatively transcribed form of Ron, known as short-form Ron (sfRon). Short-form Ron is generated from an alternative transcriptional start site under control of a second promoter within exon 10 of the *RON* gene. The sfRon protein is translated in-frame but lacks the N-terminus of Ron, including the ligand-binding domain. Thus, sfRon organizes into a constitutively-active receptor with ligand-independent activity [3]. *In vitro* and *in vivo* studies suggest that sfRon expression has more potent biological outcomes than those observed with full-length Ron expression, presumably because of the constitutive kinase activity of the sfRon protein [3]. The presence of sfRon in various tumor types has been previously noted [4, 5]; however, its function remains poorly understood. Our previous studies revealed that, in breast cancer, the major active Ron isoform in tumors from patients is short-form Ron, rather than full-length Ron. We have determined that sfRon plays a significant role in the aggressiveness of breast cancer *in vivo* by dramatically promoting tumor growth and metastasis [3].

sfRon is of particular interest in tumorigenesis, and the mouse ortholog of sfRon (also known as sfStk), has a clear role in cancer susceptibility. Naturally occurring genetic polymorphisms in the second promoter of the *stk* gene prevents production of mouse sfRon and induces resistance to Friend Virus (Fv)-induced erythroleukemia [6]. Interestingly, mouse strains that are unable to produce sfRon are also resistant to other malignancies [6, 7]. However, despite its clear role in progression of breast tumors in human xenograft models [3], no information is available so far on the role of sfRon in initiation or progression of other cancers, or whether sfRon is involved in human cancer susceptibility.

To address these questions, we conducted a comprehensive study aimed to determine the role of sfRon in tumorigenesis of various cancer types in mice. Using a carcinogen-induced tumor model, we observed that loss of sfRon expression completely protected mice from ovarian cancer. This discovery provoked further exploration of the role of sfRon in human ovarian cancer. We show that sfRon is expressed in several subtypes of human ovarian cancer, which is in contrast to its absence in healthy ovary tissue. In particular, sfRon is highly expressed in high-grade serous ovarian cancer (HG-SOC), the most prevalent and deadly subtype of ovarian cancer. We also report that ectopic expression of sfRon in OVCAR3 cells (hereafter called OVCAR3-sfRon) leads to phenotypic and functional changes associated with epithelial-to-mesenchymal transition (EMT), activation of the PI3K pathway, activation of PDK1 signaling cascade and inhibition of the MAPK pathway. Our data demonstrate that sfRon confers a more aggressive cancer phenotype *in vitro*, which is characterized by increased proliferation, increased migration, and decreased adhesion to tissue culture surfaces. Importantly, the aggressive behavior noted *in vitro* with OVCAR3-sfRon cells was also reflected in the *in vivo* studies. Tumors derived from OVCAR3-sfRon cells exhibit significantly more robust growth and metastasis within the abdominal cavity when compared with their parental sfRon negative counterparts. This work suggests for the first time that sfRon is involved in ovarian cancer initiation and progression, and suggests that inhibition of sfRon kinase activity could be considered as a strategy to combat ovarian cancer in humans.

## RESULTS

### sfRon expression is associated with susceptibility to various tumor types

**Figure 1:**
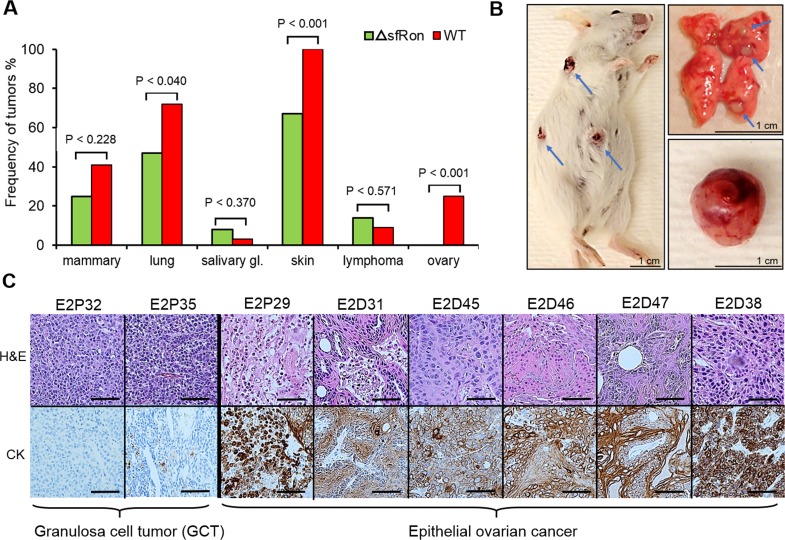
Frequency of DMBA-induced tumors in mice A. The sfRon deficient mice (ΔsfRon) (n=36) and wild type (WT) control mice on FVB/NJ background (n=32) were treated weekly with 1 mg of DMBA for 6 weeks. Lack of sfRon completely protected mice from ovarian cancer. The frequency of ovarian cancer is 8/32 (25%) in WT mice vs 0/36 (0%) in ΔsfRon mice. B. Gross observation of WT mouse bearing skin tumors (papillomas) and appearance of developing tumors in dissected lungs and ovary. The frequency of these malignancies was significantly increased in the cohort of WT mice vs ΔsfRon mice. C. H&E (upper panel) and cytokeratin (lower panel) stain of tumor sections from DMBA induced ovarian tumors in ΔsfRon mice. DMBA treatment resulted in development of various ovarian tumors in ΔsfRon mice such as granulosa cell tumor (E2P32 and E2P35); poorly differentiated carcinoma (E2P29 and E2D38); squamous cell carcinoma (E2D31); adenosquamous cell carcinoma (E2D45, E2D46 and E2D47). The images were taken at × 20 magnification and the scale bars represent 100 μm.

Our previous work revealed that sfRon is an important contributor to breast cancer pathogenesis [3, 8]. To complement our studies focused on the role of sfRon in breast tumor progression and metastasis, we investigated the requirement for endogenous sfRon in the initiation of breast and other cancers. To determine the causal role of sfRon in our studies, we utilized sfRon-deficient mice (ΔsfRon), which are engineered to be specifically unable to produce sfRon through replacement of the mouse *Ron/sfRon* gene with full-length *Ron* cDNA under control of the endogenous locus [9]. We used a classic approach, whereby we exposed ΔsfRon mice or wild type (WT) controls on a matched genetic background, FVB/NJ to 7,12-dimethylbenz[α]anthracene (DMBA), which induces a myriad of malignant tumors [10]. We treated cohorts of ΔsfRon or WT mice (n=36 and n=32, respectively) with DMBA weekly for six weeks. As expected, both strains of mice developed many different malignancies within 2-7 months after DMBA treatment. We observed development of overall higher numbers of different types of tumors in WT mice vs ΔsfRon mice (an average of 3.2 tumors per mouse in controls vs 1.6 tumors per mouse in ΔsfRon group; P = 0.0003), which suggests a role for sfRon in promoting tumorigenesis. Analysis of incidence rates for various cancers revealed that among ΔsfRon mice 25% had mammary tumors; 47% had lung cancer; 8% had salivary gland tumors; 67% had skin cancer; 14% had lymphoma and 0% had ovarian cancer. In WT mice, 41% of animals developed mammary tumors; 72% had lung cancer; 3% had salivary gland tumors; 100% had skin cancer; 9% had lymphoma; and 25% of animals had ovarian cancer (Fig. 1A, B). Thus, we noted significantly decreased tumor formation in ovaries, skin and lungs of ΔsfRon mice, while other tumor types were not significantly different between cohorts (Fig. 1B). In particular, lack of sfRon appeared to completely protect the mice from ovarian cancer (Fig. 1A).

### Lack of sfRon expression protects mice from ovarian tumor

The frequency of ovarian tumors was 8/32 (25%) in wild-type mice vs. 0/36 (0%) in ΔsfRon mice (P = 0.001). There was no difference in the length of the experiment between cohorts, so lack of ovarian tumor development in ΔsfRon mice could not be explained by early death of mice in that group (P = 0.067). To our knowledge, this study is the first to implicate sfRon in the initial development of ovarian tumors. The histology of the DMBA-induced ovarian tumors in WT mice was examined by hematoxylin and eosin (H&E) staining. To determine the epithelial nature of DMBA-induced ovarian tumors, we also performed immunohistochemical (IHC) analysis on tumor sections using a broad spectrum cytokeratin (pan-CK) antibody. The IHC analysis revealed that 75% of the DMBA-induced ovarian tumors were of epithelial origin, comprising squamous cell carcinomas, adenosquamous cell carcinomas, and poorly differentiated carcinomas. 25% of the tumors were granulosa cell tumors (Fig. 1C). Necropsy revealed that the epithelial ovarian tumors extensively invaded the peritoneal cavity, while the granulosa cell tumors were mostly confined to the ovary; notably, consistent with the common tumor behavior observed in human subjects with these distinct subtypes of ovarian cancer.

### sfRon is robustly expressed in human ovarian tumors

**Figure 2:**
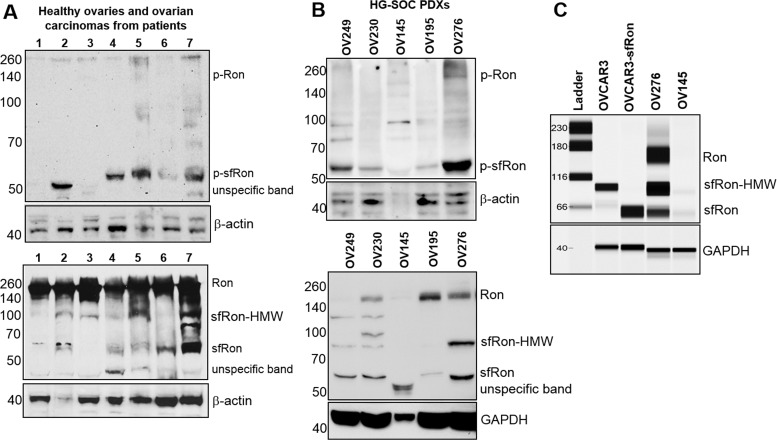
The expression and activity of Ron receptor isoforms in primary tumors and high-grade serous ovarian (HG-SOC) PDXs A. Upper panel displays western blot analysis of tumor lysates from patients assayed for phosphorylated (active) sfRon and Ron. Lower panel shows western blot analysis of total levels of Ron isoforms. The higher molecular weight sfRon bands (sfRon-HMW), which are putative, posttranslationally modified sfRon forms were also noted. The blots were stripped and re-probed for β-actin. Lanes represent: healthy ovary (1, 2, 3); ovarian adenocarcinoma (4); carcinosarcoma (5); endometrioid adenocarcinoma (6) and HG-SOC (7). B. Upper panel displays western blot analysis of tumor lysates from HG-SOC PDXs assayed for phosphorylated Ron isoforms. Lower panel shows western blot analysis of total levels of Ron isoforms. The blots were stripped and re-probed for β-actin or GAPDH. C. The expression of Ron isoforms was assessed by WES capillary electrophoresis-based protein assay in OVCAR3-sfRon cell line engineered to express sfRon vs. parental OVACR3 cells and compared with sfRon positive or negative HG-SOC PDXs. GAPDH was used as loading control.

Our studies indicated that sfRon may represent an unappreciated driver of ovarian tumorigenesis, so we investigated it further using primary ovarian cancer specimens from patients. To assess the abundance and activity of sfRon protein, we performed Western blot analysis on primary human ovarian tumors, healthy ovarian tissue and patient-derived xenografts (PDXs) of high-grade serous carcinoma histology (HG-SOC). These studies revealed that sfRon protein is expressed and active in several different histological types of human ovarian cancer and is commonly expressed in HG-SOC PDXs (Fig. 2A, B). Moreover, we evaluated the expression of Ron isoforms in 3 of our newly developed HG-SOC PDX tumor models, which have also been immunohistochemically characterized for the expression of commonly used markers for HG-SOC subtype such as pan-cytokeratin (CK), PAX8 and WT1 (Supplementary Fig. 3). Our data indicate that, in contrast to ovarian carcinomas, sfRon expression was not detectable in healthy ovarian tissue (Fig. 2A). Based on this data, we aimed to further investigate the function of sfRon in the most common and lethal subtype of ovarian cancer, HG-SOC [11].

**Figure 3:**
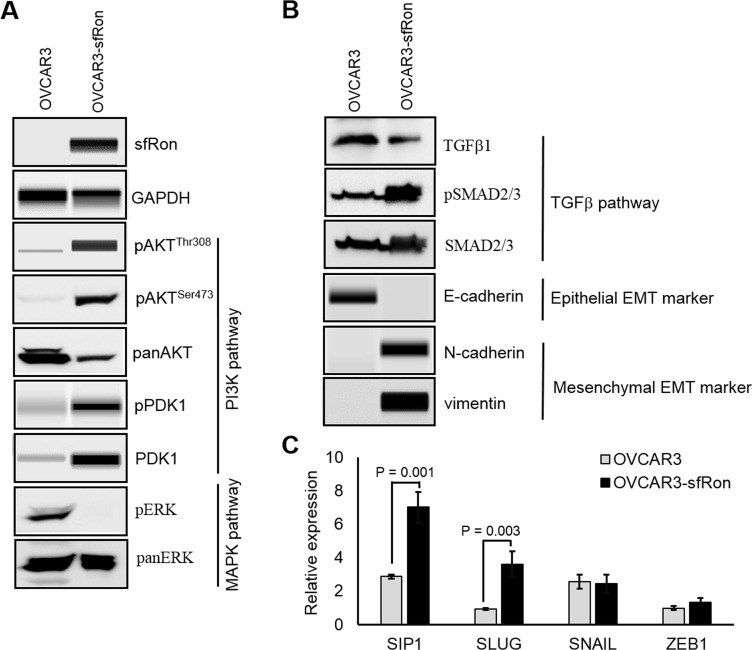
The sfRon signaling pathway in ovarian cancer A. Analysis of the effects of sfRon expression on the activity of PI3K and MAPK signaling pathways downstream from sfRon. Indicated proteins were detected by WES capillary electrophoresis-based protein assay (sfRon, GAPDH, pAKT^Thr308^, pPDK1, PDK1) or standard Western Blot (pAKT^Ser473^, pERK, panAKT, panERK). Whole Blots or WES images are shown in Supplementary Fig. 1. B. Analysis of the activity of TGFβ pathway and the expression of EMT marker proteins in OVCAR3-sfRon vs parental OVCAR3 cells. Indicated proteins were detected by standard Western Blot (TGFβ1, pSMAD2/3, SMAD2/3) or by WES capillary electrophoresis-based protein assay (E-cadherin, N-cadherin, vimentin). Whole Blots or WES images are shown in Supplementary Fig. 2. C. The qRT-PCR analysis of EMT related transcription factors such as SIP1, SLUG, SNAIL and ZEB1.

### sfRon expression induces epithelial-to-mesenchymal transition (EMT)

**Figure 4:**
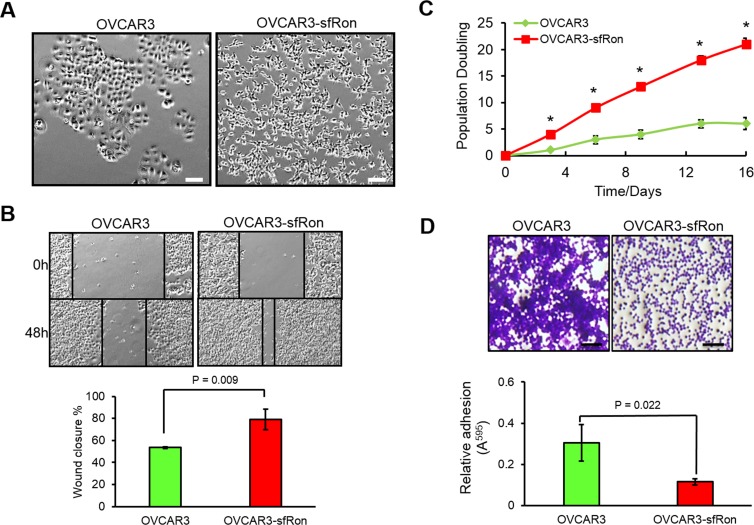
The effects of sfRon expression in OVCAR3 cells in vitro A. Phase-contrast micrograph illustrating altered morphology of OVCAR3 cells after introduction of sfRon compared to those without sfRon expression. Scale bars represent 100 μm. B. Images of wound healing assay. The effect of sfRon expression on migration of OVCAR3 cells was assessed by recover of the scratch. The area of the wound was measured, and % of wound closure was compared between OVCAR3 and OVCAR3-sfRon cells after 48h. C. The graph represents the effect of sfRon expression on OVCAR3 vs OVCAR3-sfRon cells proliferation assessed by 3T5 cell growth assay. Each point on the curve is an average measurement of cell count from a three plates followed over the course of the experiment. D. Changes in adhesion of OVCAR3 cells after introduction of sfRon were evaluated by cell adhesion assay. The adherent cells were stained with 0.5% crystal violet, imaged and quantified at OD 595 nm after extraction. Scale bars represent 100 μm.

To study the role of sfRon in high-grade serous ovarian cancer pathogenesis we utilized the NIH-OVCAR3 cell line (we refer to this cell line as OVCAR3) [12]. Assessment of sfRon expression in OVCAR3 cell line revealed that these cells do not express appreciable levels of sfRon, in contrast to HG-SOC PDXs (Fig. 2C). It is well recognized that cell lines only partially recapitulate the genetic features and heterogeneity of ovarian tumors in patients [13]. In fact, loss of endogenous sfRon expression is a feature of many cancer cell lines in culture, despite its robust expression in actual human tumors [3]. In order to study the role of sfRon in pathogenesis of high-grade serous ovarian cancer, OVCAR3 cells were engineered to stably express sfRon (Fig. 2C). The sfRon protein expression in OVCAR3 cells (OVCAR3-sfRon) was verified by WES immunoassay and was comparable to endogenous levels of sfRon found in HG-SOC PDX models (Fig. 2C). Next, we asked whether introduction of sfRon into OVCAR3 cancer cells would alter their behavior. After culturing OVCAR3-sfRon cells *in vitro* for 3 weeks, we observed that cells underwent distinct morphological changes consistent with a shift from an epithelial to a mesenchymal phenotype (Fig. 4A). The epithelial-to-mesenchymal transition (EMT) is an integral process in development, wound healing and stem cell behavior, and contributes pathologically to fibrosis, cancer progression, and therapy resistance [14-16]. In order to verify the EMT in OVCAR3-sfRon cells and gain an insight into the potential signaling molecules that are involved in this process, we performed WES immunoassay analysis on OVCAR3 and OVCAR3-sfRon cell lysates. We evaluated the expression of E-cadherin, N-cadherin and vimentin, which are a hallmarks of EMT. We observed decreased expression of the epithelial marker E-cadherin and increased expression of the mesenchymal markers N-cadherin and vimentin in OVCAR3-sfRon cells when compared with parental OVCAR3 cells (Fig. 3B). The EMT is a very complex process controlled by various transcriptional regulators through different signaling pathways. EMT-promoting signaling pathways appear to be responsible for expression and activation of some EMT master regulators, including Slug, SIP1, SNAIL and zinc-finger E-box-binding (ZEB) transcription factors. These master regulators can act pleiotropically to choreograph the complex EMT program [14, 17]. We subsequently quantified the mRNA expression patterns of several EMT-inducing transcription factors using qRT-PCR. The results demonstrated significantly increased expression of EMT master regulators such as Slug and SIP1 in OVCAR3-sfRon cells when compared with parental OVCAR3 cell line (Fig. 3C), which confirmed the EMT transition of OVCAR3-sfRon cells.

Among different factors involved in EMT initiation, transforming growth factor-β (TGFβ) signaling has a notable role; however, it has been demonstrated that other signaling cascades such as PI3K or MAPK, downstream of receptor tyrosine kinases also play an essential role in this trans-differentiation process [14, 17]. We assessed the activity of PI3K, MAPK and TGFβ signaling pathways in OVCAR3 and OVCAR3-sfRon cell line to reveal a potential signaling cascade responsible for induction of the EMT in sfRon expressing cells. Our data shows that OVCAR3-sfRon cell line is characterized by activation of the PI3K signaling network, as assessed by increased phosphorylation of AKT (AKT^Thr308^, AKT^Ser473^) and increased expression and phosphorylation of PDK1 (PDK1^Ser241^). In contrast, we noted loss of MAPK pathway signaling, as determined by lack of p-ERK1/2 expression (Fig. 3A). The examination of the activity of TGFβ signaling pathway in OVCAR3 vs OVCAR3-sfRon cells revealed that the pathway is active in both cell lines, which is reflected in the presence of TGFβ1 protein and phosphorylation of SMAD2/3 proteins downstream from TGFβ receptors (Fig. 3B). Overall, these data suggest that the transition to a mesenchymal phenotype after introduction of sfRon into OVCAR3 cells is likely due to strong activation of the PI3K pathway. These data are consistent with our previous findings in breast cancer cells, in which PI3K signaling downstream of sfRon was required for EMT [3].

### sfRon expression enhances proliferation and migration of ovarian cancer cells

Since the acquisition of a mesenchymal phenotype has been proposed as the critical mechanism for a tumor progression, invasion into surrounding tissues and subsequent systemic spread of cancer cells [17], we performed various phenotypic cell-based assays to evaluate the potential tumor promoting effects of sfRon expression in OVCAR3 cells. First, 3T5 cell proliferation assay was performed to measure cumulative population doublings. The results showed that OVCAR3-sfRon cells proliferate significantly faster than the parental OVCAR3 cells. After 16 days, OVCAR3-sfRon cells reached 21 population doublings. In contrast, parental OVCAR3 cells reached only 6 population doublings over the same period of time (Fig. 4C). Next, we evaluated the adhesion and migration properties of the cells. One key feature of an epithelial ovarian cancer is its ability to detach from the primary tumor site and “implant” diffusely along all peritoneal surfaces resulting in widespread tumor growth with related organ dysfunction and ultimately death. Our results show that OVCAR3-sfRon cells have significantly diminished adhesive properties compared to parental cells (OVCAR3-sfRon cells were 2.6 times less adhesive; Fig. 4D). This suggests that sfRon expression confers invasive abilities to ovarian cancer cells. To address the impact of sfRon expression on the motility of OVCAR3 cells we performed a scratch-based wound healing assay. After OVCAR3 and OVCAR3-sfRon cells reached 70% confluence, scratches were made in a cell monolayer and we tracked migration of individual cells in the leading edge of the scratch until the wound gap was closed. We measured and quantified the gap distance of the wound 48h after cultures were scratched. We observed that the scratch closure rate was significantly increased in OVCAR3-sfRon cells vs OVCAR3 cells (P = 0.009). After 48h, OVCAR3-sfRon cells had closed the wound gap by 79%, and OVCAR3 cells closed the wound gap by 54% (Fig. 4B). Thus our *in vitro* observations of the effect of sfRon expression in OVCAR3 cells revealed that sfRon causes ovarian cancer cells to acquire aggressive, invasive phenotypes comprising EMT, increased proliferation, increased migration and decreased adhesion.

### sfRon expression promotes progression of ovarian cancer in vivo

**Figure 5:**
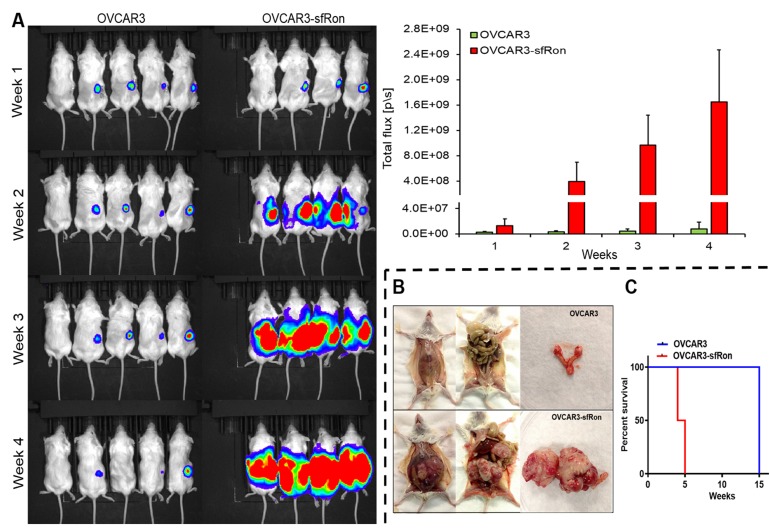
The effects of sfRon expression in OVCAR3 cells in vivo A. Bioluminescence imaging of luciferase activity in mice bearing orthotopic OVCAR3 or OVCAR3-sfRon tumors. Luciferase expression was measured weekly for 4 weeks after injection of 5×10^5^ cells into ovary of NSG mice, which is quantified in graph (right panel). B. Representative images of mice from the two groups showing the progression of orthotopic ovarian tumor 5 weeks after tumor cells inoculation. C. Survival curves of mice bearing OVCAR3 or OVCAR3-sfRon tumors. Mice in OVCAR3-sfRon group were euthanized at week 5 due to advanced tumor burden, while mice bearing OVCAR3 tumors lived for 15 weeks.

To determine whether the aggressive phenotype of OVCAR3-sfRon cells *in vitro* was related to tumor aggressiveness *in vivo*, we orthotopically implanted 5 × 10^5^ luciferase-expressing OVCAR3 or OVCAR3-sfRon cells into ovaries of NSG mice. Beginning one week after cell implantation, we monitored tumor progression weekly by quantitative IVIS bioluminescence imaging, whereby the bioluminescent signal is expressed in photons per second (photon flux) and displayed as an intensity map. Photon flux from the tumor is proportional to the number of live cells expressing luciferase, so bioluminescence is a surrogate measure of tumor burden [18]. We observed that mice implanted with OVCAR3-sfRon cells exhibited rapid tumor growth, and required euthanasia 4-5 weeks post-implantation due to severe tumor burden. In contrast, mice implanted with parental OVCAR3 cells showed significantly slower tumor progression, reaching the experimental endpoint around week 15 (Fig. 5). At week 5, we euthanized one mouse from each group and performed necropsy to macroscopically evaluate tumor progression. At this time point, a mouse implanted with OVCAR3-sfRon cells had developed a huge tumor in the ovary, which had already spread into the abdominal cavity. In contrast, a mouse implanted with parental OVCAR3 cells had developed a tumor in ovary that was detectable by IVIS imaging, but was not visible to the naked eye (Fig. 5B). Finally, we compared mouse survival among cohorts of animals bearing OVCAR3 or OVCAR3-sfRon tumors. We found significant differences in mouse survival between the cohorts: OVCAR3-sfRon tumor bearing mice lived only 4-5 weeks, while OVCAR3 tumor bearing mice lived 15 weeks (Fig. 5C).

## DISCUSSION

The data presented here reveal the role of sfRon in ovarian cancer development and progression. The study of DMBA-induced mouse tumors using sfRon deficient (ΔsfRon) mice vs control mice resulted in the striking discovery that lack of sfRon expression completely protected mice from ovarian cancer (P = 0.001). To the best of our knowledge, this study is the first to demonstrate the critical importance of sfRon expression in development of ovarian tumors in mice. Upon pathological analysis, 75% of the DMBA-induced ovarian tumors in our study appeared to have originated from ovarian surface epithelium, whereas the 25% remaining malignancies were granulosa cell tumors. These findings can be compared with other reports of female mice treated with DMBA, in which 14% of animals developed ovarian cancers; two thirds of these were serous cystadenomas of epithelial origin, and the rest were granulosa cell tumors [19].

In our study, we also found a high incidence of lung tumors in WT mice, and fewer lung tumors in ΔsfRon mice treated with DMBA (72% and 47%, respectively). It has been previously reported that the most common spontaneous tumor found in aging FVB/NJ mice is a lung cancer [20]. In addition, other studies showed that among Ron transcripts, sfRon is the major one in the lung [21] and sfRon protein is constitutively tyrosine-phosphorylated in the lung cancer cells [5]. Taken together, these data indicate that sfRon might also play a role in lung cancer and is worthy of further investigation.

Interestingly, despite a clear role for sfRon in breast cancer progression and metastasis, we did not see a significant decrease in mammary tumor development in ΔsfRon mice treated with DMBA. It is possible that sfRon is specifically involved in tumor progression in the breast (rather than initiation); however, a role for sfRon in DMBA-independent tumor initiation cannot be ruled out without examination of more animals.

The fact that mice lacking sfRon were protected from ovarian tumors in our experiments prompted us to evaluate the expression of sfRon in human ovarian tumors and study the role of this protein in ovarian cancer biology. Here, we have demonstrated that sfRon is expressed in various subtypes of ovarian cancer such as ovarian adenocarcinoma, carcinosarcoma, endometrioid adenocarcinoma and high-grade serous ovarian cancer (HG-SOC), compared to undetectable expression in healthy ovary. We recognize that the normal ovary is not the best healthy control tissue for all ovarian carcinomas. However, to the best of our knowledge sfRon protein expression has never been reported in healthy ovaries, fallopian tubes or endometrium, which reinforce the idea that the expression of sfRon might be an important factor in ovarian tumor progression (especially the high-grade serous subtype, where sfRon is robustly expressed). It is noteworthy that in a previous study, short-form Ron transcript was detected in normal ovaries by Bardella at. al. However, the Authors were unable to detect sfRon protein expression in healthy ovaries by immunohistochemistry, which is consistent with our observation that normal ovaries lack sfRon protein expression [4]. The selective presence of sfRon protein in tumors could be explained by several potential mechanisms, including inappropriate stabilization of sfRon mRNA, increased translation of sfRon mRNA, and/or improved protein stability. Our data also show that full-length Ron protein seems to be expressed in healthy ovaries (Fig. 2A), which is in agreement with previous findings that show presence of full-length Ron receptor (but not short-form Ron) in healthy breast epithelium [3].

Since we noted that sfRon is abundantly expressed in the most common and aggressive subtype of epithelial ovarian cancers, HG-SOC, we focused our efforts on understanding the role of sfRon in pathogenesis of this cancer. Our current study demonstrated that exogenous expression of the truncated isoform of Ron receptor (sfRon) has a profound effect on proliferation and invasiveness of ovarian cancer cells. These results are consistent with observations published by Merlin et. al.. In their work, the Authors described the unleashing of the tumorigenic and invasive potential of a constitutively active, truncated form of the MET receptor, which belongs to the same family of receptors as Ron [22].

Our data show that engineered expression of sfRon in OVCAR3 cells resulted in activation of PI3K and PDK1 pathway; inhibition of MAPK pathway; EMT; increased proliferation; increased migration; and decreased adhesion. The ability of sfRon to induce EMT, activate PI3K pathway and block MAPK pathway was previously reported in MCF7 breast cancer cells by our group [3]. In addition, in previous work, we demonstrated the requirement of the PI3K pathway for sfRon tumor promoting function, and that blocking the interaction between sfRon and PI3K thoroughly abrogated the ability of sfRon to confer aggressive tumor behavior *in vitro* [3]. However, which PI3K downstream targets are involved in the progression of sfRon expressing tumors has not yet been determined.

Although AKT is recognized to be one of the key proteins regulating growth and proliferation of cancer cells, there is increasing evidence that AKT-independent pathways, downstream of PI3K, may also play a crucial role in driving tumor progression [23, 24]. In fact, our previous work revealed that although sfRon-driven breast tumor progression is strongly associated with activation of PI3K signaling, it does not appear to be through the AKT/mTOR pathway, because inhibition of AKT or mTOR was not able to suppress any of the tumor promoting functions of sfRon [3]. These finding indicate that factors downstream from PI3K pathway, other than AKT/mTOR, may be responsible for promoting aggressive tumor behavior [3]. In this respect, the existence of activated and strongly expressed PDK1 protein in OVCAR3-sfRon cells with no expression of this protein in OVCAR3 cells clearly indicates a potential role for PDK1 in progression of ovarian cancers expressing sfRon. PDK1 is a transducer of PI3K signaling that functions as a central hub for many crucial cellular signaling pathways. As a serine/threonine protein kinase, PDK1 activates a large number of proteins of the conserved AGC kinase superfamily, including AKT, some PKC isoforms, S6K, RSK, SGK and ROCK1 [25, 26]. In recent years PDK1 has been an emerging target of importance in pathogenesis of various tumor types [24, 25, 27-30]. Importantly, a role for PDK1 in ovarian cancer progression has been recently demonstrated, where authors showed that normal ovaries show no significant levels of PDK1, but enhanced expression of PDK1 was observed in borderline and low- to high-grade ovarian tumors [31]. Several other studies demonstrated that expression of PDK1 induces EMT [30], anchorage-independent growth *in vitro* [29, 30], increased proliferation [28], migration and invasion of cancer cells [27-30, 32]. These are also the prominent, tumor promoting features acquired by OVCAR3 cells after introduction of sfRon, which is associated with strong activation of PDK1 pathway in these cells.

Our current study also demonstrated the profound effect of sfRon expression on ovarian cancer growth and spreading to abdominal cavity *in vivo*, which is in agreement with previous work investigating the *in vivo* role of sfRon in breast cancer [3], and reinforce the idea that sfRon may be a promising therapeutic target for high-grade serous ovarian cancer.

In summary, this work showed a previously unappreciated and important role for sfRon in ovarian cancer development and progression. Further studies designed to determine the specific mechanism by which sfRon induces high-grade serous ovarian cancer progression, with a strong focus on the role of PDK1 in this process, are warranted. An increasing number of studies suggest that PDK1 inhibitors may be useful to prevent cancer progression and abnormal tissue dissemination [27, 31, 33]. The recent discovery of more potent and selective PDK1 inhibitors [34, 35] and Ron inhibitors [36, 37], and their forthcoming tests in tumor models will be instrumental to understand the significance of sfRon and PDK1 inhibition in high-grade serous ovarian cancer.

## MATERIALS AND METHODS

### Animal Studies

### DMBA treatment of FVB/NJ and ΔsfRon mice

All experimental procedures involving FVB/NJ and ΔsfRon mice were approved by the Institutional Animal Care and Use Committee (IACUC) of Huntsman Cancer Institute at University of Utah. FVB/NJ mice were purchased from the Jackson Laboratory (Bar Harbor, ME). ΔsfRon mice were generous gift from Dr. Susan E. Waltz (University of Cincinnati Cancer Institute) and described elsewhere [9]. Virgin female FVB/NJ mice (n = 32) and ΔsfRon mice (n = 36) were treated weekly for 6 weeks with 1 mg of DMBA dissolved in olive oil by oral gavage beginning at 6-8 weeks of age. Beginning 8 weeks after the final dose, mice began to develop evidence of tumors and by 28 weeks all mice had developed tumors. Gross clinical examination of mice was done weekly to monitor body weight, development and progression of different tumors and evaluation of symptoms of physical distress or illness. Mice showing severe tumor burden where euthanized by CO_2_ inhalation and necropsied. Tissues were fixed in 10% phosphate-buffered formalin and embedded in paraffin for histological analysis or flash frozen for molecular and biochemical studies.

### OVCAR3 and OVCAR3-sfRon xenograft studies

All experimental procedures involving NSG mice were approved by the Institutional Animal Care and Use Committee (IACUC) of the Oklahoma Medical Research Foundation. NSG mice were purchased from the Jackson Laboratory (Bar Harbor, ME). 5×10^5^ luciferase expressing OVCAR3 or OVCAR3-sfRon cells in 50 μl of 1X PBS were orthotopically injected into the right ovary of animals (via the intra-ovarian bursa, IB) using a 30 gauge needle. Mice were monitored weekly for body weight, development and progression of ovarian tumors, and any symptoms of physical distress or illness. One week after tumor cells inoculation mice showed evidence of developing tumor in ovary. Starting at week 1 animals were imaged weekly for luciferase activity until they required euthanasia due to severe tumor burden. Bioluminescence imaging was performed using a cooled CCD camera (Xenogen IVIS, Xenogen, Alameda, CA), coupled to the LivingImage acquisition and analysis software (Xenogen Corp.). Before imaging both cohorts of mice received intraperitoneal (IP) injections of luciferin (Gold Biotechnology, Inc., St. Louis, MO) in a dose of 150 mg/kg of body weight. Bioluminescence was measured over an integration time of 1 min, images were acquired using LivingImage software, and quantified as bioluminescence radiance (photon flux). Peritoneal luciferase activity was correlated with the distribution and size of ovarian tumors. Over the course of experiment animals were euthanized when they showed advanced tumor burden. At necropsy mice underwent visual inspection of peritoneal tumor load.

### Histology

Upon necropsy, harvested tumors were fixed in 10% neutral buffered formalin, paraffin embedded, and hematoxylin-eosin (H&E) stained according to our standard protocols [38]. DMBA-induced ovarian tumors were analyzed by Immunohistochemistry (IHC) for expression of the epithelial marker cytokeratin (1:400, DAKO, Carpinteria, CA, #Z0622). Staining was visualized by 3,3-diaminobenzidine, with hematoxylin as a counter-stain [38]. Slides were imaged on an Olympus Bx50 microscope with a Canon EOS Rebel XSI camera using EOS imaging software. Sections stained with H&E and cytokeratin were subjected to a blind review by a pathologist.

### Cell lines and culture

OVCAR3 cells expressing sfRon (OVCAR3-sfRon) were generated in similar way as MCF7-sfRon cells described elsewhere (4). OVCAR3 cell lines were cultured and maintained in RPMI 1640 medium (Gibco) with 10% fetal bovine serum (Gibco) at 37°C in a 5% CO_2_ incubator. Our parental OVCAR3 (NIH-OVCAR3) cell line was purchased from ATCC (NIH: OVCAR-3, ATCC® HTB-161™) on 4/10/2012. ATCC authenticates their cell lines by short tandem repeat (STR) profiling analysis. To ensure the identity and validity of our cell lines and to prevent potential problems associated with cell culture, such as cell line misidentification, contamination and genetic drift, we purchase cell lines form validated, reliable source (e.g. ATCC) and cryopreserve 20 1 ml vials of each cell line at low passage (passage 1-3). The vials of low passage cell lines are kept protected in lab cell line bank and distributed to lab members according to the experimental needs. Recently, we performed authentication of several cell lines used in our recent published work (Bieniasz et. al., 2015) such as MCF7 and MCF7-sfRon, which have been in use in our lab for 8 years. Authentication performed by ATCC revealed that all tested cell lines showed 100% match to the original cell lines from which they were derived.

### Ovarian tumor tissues and HG-SOC PDXs

Ovarian tumor tissue samples were collected from patients who provided informed consent at Huntsman Cancer Hospital/University of Utah under an approved institutional review board protocol. HG-SOC PDXs were acquired from StemCentrx (San Francisco, CA).

### Immunoblotting

Cells or tumors were lysed in Buffer B (25 mM Tris-HCl, pH 7.5, 0.42 M NaCl, 1.5 mM MgCl2, 0.5 mM EDTA, 1 mM DTT, 25% sucrose, 1 mM Na3VO4, and 1X protease inhibitor cocktail) on ice for 15 min, followed by centrifugal clearing at 4oC for 10 min at 10,000 rpm to recover whole cell lysates.

For Western Blotting cellular proteins (100 μg whole cell lysate) were separated by 10% SDS-PAGE under reduced conditions and transferred to PVDF membranes (Millipore Co., Billerica, MA). Primary antibodies used were: anti-pRon (1:400, #AF1947) from R&D; anti-Ron (1:500, # sc-322); anti-GAPDH (1:2000, #sc-25778); anti-TGFβ1 (1:500, #sc-146); anti-pSMAD2/3 Ser^423/425^ (1:400, #sc-11769); anti-SMAD2/3 (1:800, #sc-8332) from Santa Cruz Biotechnology (Santa Cruz, CA); anti-β-actin (1:1000, #ab6276) from Abcam; anti-phospho Akt Ser^473^ (1:500, #9271); anti-pan Akt (1:1000, #4691); anti-pERK Thr^202^/Tyr^204^ (1:1000, #4370S); anti-pan ERK (1:2000, #4695) from Cell Signaling Technology. Anti-rabbit or anti-mouse secondary antibodies, conjugated with horse radish peroxidase (Santa Cruz, CA) were applied, and specific bands were visualized using Western Lightning® Plus- ECL (PerkinElmer). Levels of chemiluminescence were captured and quantified with the ChemiDoc XRS system with Image Lab Software.

For analysis of proteins using a capillary electrophoresis-based protein analysis system (WES; ProteinSimple, San Jose, CA), cellular proteins (0.5 mg/ml) were separated and visualized using the standard instrument protocol. Primary antibodies used were: anti-Ron (1:25, # sc-322); GAPDH (1:300, #sc-25778); vimentin (1:10, #sc-5565) from Santa Cruz Biotechnology (Santa Cruz, CA); anti-phospho Akt Thr^308^ (1:10, #13038); anti-pPDK1 Ser^241^ (1:10, #3061), anti-PDK1 (1:10, #3062) from Cell Signaling Technology; E-cadherin (1:10, #610182), N-cadherin (1:10, #610920) from BD Bioscience. Anti-rabbit secondary antibodies were included in the Wes-Rabbit (12-230 kDa) Master Kit (# PS-MK14, ProteinSimple, San Jose, CA).

### qRT-PCR

Total RNA was extracted with the Qiagen RNeasy Mini kit (Qiagen, Valencia, CA), according to the Manufacturer's protocol. cDNA was synthesized using iScript cDNA synthesis kit (#1708840, Bio-Rad, Hercules, CA) following the manufacturer's instructions. A 20 μL reaction volume was prepared with 1 μg purified RNA. cDNA was diluted 1:20 in nuclease-free water and 1μL was analyzed in triplicates by real-time PCR using the LightCycler 96 System (Roche) and PowerUp™ SYBR® Green Master Mix (#A25742, Thermo Fisher Scientific), with 0.2 μM of each primer in a total volume of 25 μL reaction mixture. Primers were ordered from Integrated DNA Technologies, Inc. (IDT) (Coralville, IA). Primers were based on published sequences or designed using Prime 3 software (Prime 3), which are listed as the following sequences: forward primer and reverse primer. Human GAPDH: 5′ CCC TCA ACG ACC ACT TTG TC 3′ and 5′ GGG TCT ACA TGG CAA CTG TG 3′; SNAIL: 5′ CTC TGG TCT GAC CGA TGTGTC TC 3′ and 5′ ACC TGT CGG GCC CCC 3′; SLUG: 5′ GTT TTC CAG ACC CTG GTT GCT 3′ and 5′ TTC TCC CCC GTG TGA GTT CTA 3′; ZEB1: 5′ GTT CCA TTT ATG GCC TGC AT 3′ and 5′ CTG TGT TTC AAG CAC CCT CA 3′; SIP1: 5′ GCT TGG TTA GCA GGT ATTTTG ACC 3′ and 5′ CAA GAT GGC TCA TCA GCT AAATCA 3′.

### 3T3 cell proliferation assay

3T5 cell growth assay was performed by plating 5×10^5^ cells per 10 cm tissue culture plate (each cell line was set up in triplicate), followed by counting and re-plating at the same density every 3 days for 16 days. Population doubling time was calculated using the formula ln(post-3-day cell count/5×10^5^)/ln(2). The given population doubling time was added to the cumulative doubling time of the previous count.

### Adhesion assay

The assay was performed by plating in triplicates 1×10^5^ OVCAR3 or OVCAR3-sfRon cells per well of 96-well plate in serum deprived medium (0.5% FBS in RPMI 1640). Cells were incubated for 2h at 37°C followed by washing away unattached cells. Adherent cells were fixed with ice-cold methanol for 10 min and then stained with 0.5% crystal violet solution (made in 25% methanol and stored at room temperature) for 10 min in room temperature. Next, culture wells were rinsed with ddH_2_O until purple color was no longer coming off while rinsing and culture plates were dried overnight. Dried culture plates were imaged on an Olympus Bx50 microscope with a Canon EOS Rebel XSI camera using EOS imaging software and quantified at OD 595 nm after extraction using FLUOstar Omega Microplate Reader (BMG LABTECH, Cary, NC)

### Wound healing assay

Migration potential of OVCAR3 vs OVCAR3-sfRon cells was evaluated using the scratch wound healing assay. Cells were grown to 70% confluence in 6 cm culture plates. Using a 200 μl pipette tip, a wound was produced in the middle of the monolayer. The adherent monolayer was washed with 1X PBS to remove non-adherent cells and 10% FBS RPMI 1640 media was then added. At each time point (0h, 8h, 24h, 32h, 48h, 56h, 72h, 80h, 96h, 104h) cell migration in the leading edge of the scratch was tracked and imaged until wound gap was completely closed using EVOS® FL Imaging System (Thermo Fisher Scientific). 48h after cells were scratched the gap distance of the wound was quantified using ImageJ software, version 1.48v and Java 1.6.0_20 (32-bit) engine. Results of wound healing assay are expressed as percentage of wound closure of triplicate areas 48h after cells were scratched.

### Statistical analysis

All *in vitro* experiments were performed three separate times and in triplicate when applicable. Values are presented as mean ± SD. Statistical analysis of *in vitro* and *in vivo* assays was done using multiple t-test with Holm-Sidak correction. P values of less than 0.05 were considered significant. Statistical analysis was performed using GraphPad Prism 6.0 Software.

## SUPPLEMENTARY FIGURES


