# Supplementary material for: Short-form Ron is a novel determinant of ovarian cancer initiation and progression
Source: Genes Cancer. 2016 May;7(5-6):169–81. doi: 10.18632/genesandcancer.109 (PMC4979590; doi:10.18632/genesandcancer.109)
Supplement: Supplementary file 1 [file ganc-07-169-s001.pdf]

Short-form Ron is a novel determinant of ovarian cancer initiation and progression – Moxley et al

Supplementary Figures

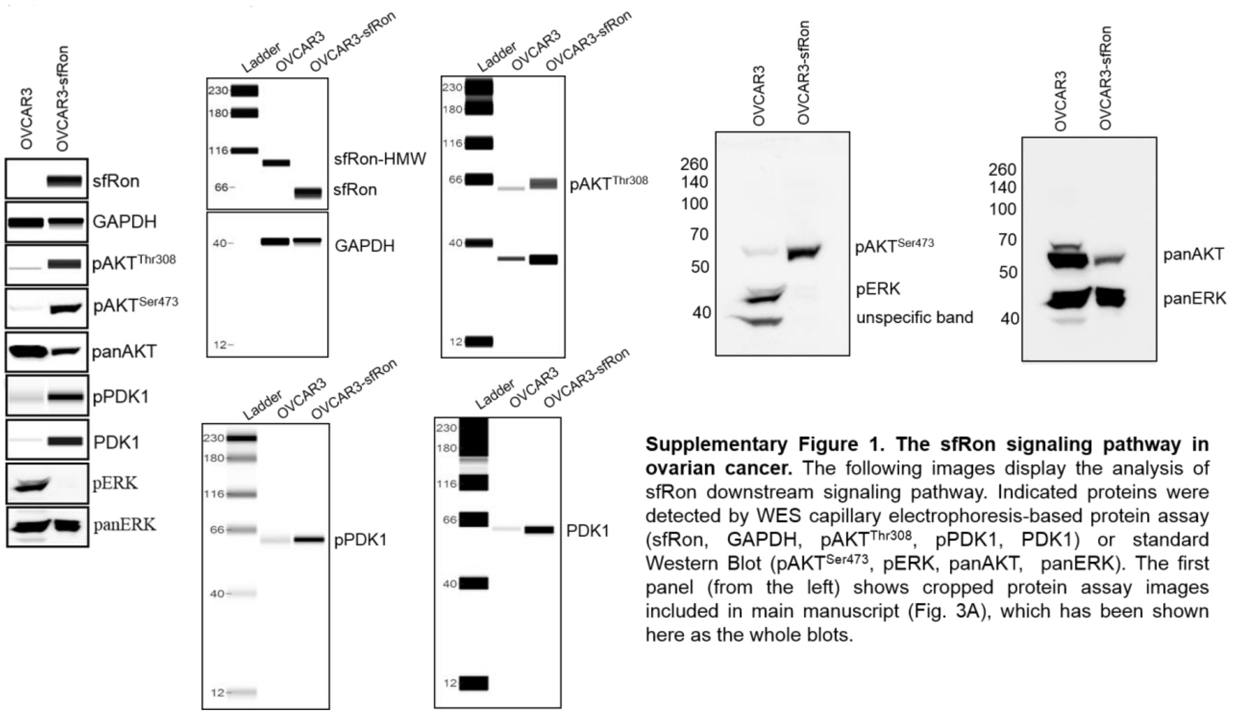

Supplementary Figure 1. The sfRon signaling pathway in ovarian cancer. The following images display the analysis of sfRon downstream signaling pathway. Indicated proteins were detected by WES capillary electrophoresis-based protein assay (sfRon, GAPDH, pAKT^{Thr308}, pPDK1, PDK1) or standard Western Blot (pAKT^{Ser473}, pERK, panAKT, panERK). The first panel (from the left) shows cropped protein assay images included in main manuscript (Fig. 3A), which has been shown here as the whole blots.

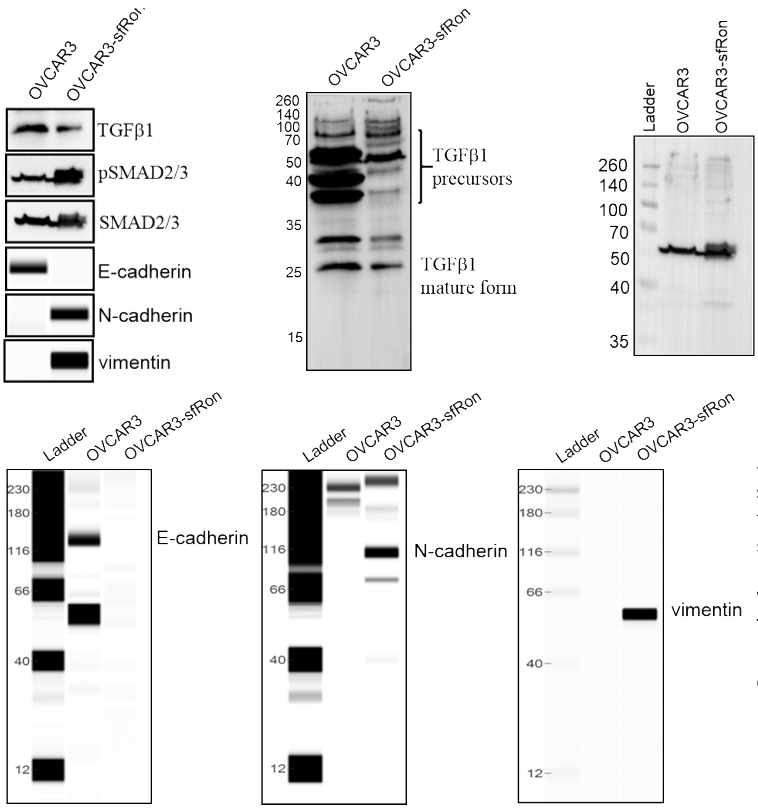

Supplementary Figure 2. The sfRon signaling pathway in ovarian cancer. The following images display the analysis of sfRon downstream signaling pathway. Indicated proteins were detected by standard Western Blot (pSMAD2/3, SMAD2/3, TGFβ1) or WES capillary electrophoresis-based protein assay (E-cadherin, N-cadherin, vimentin). The first panel (from the left) shows cropped protein assay images included in main manuscript (Fig. 3B), which has been shown here as the whole blots.

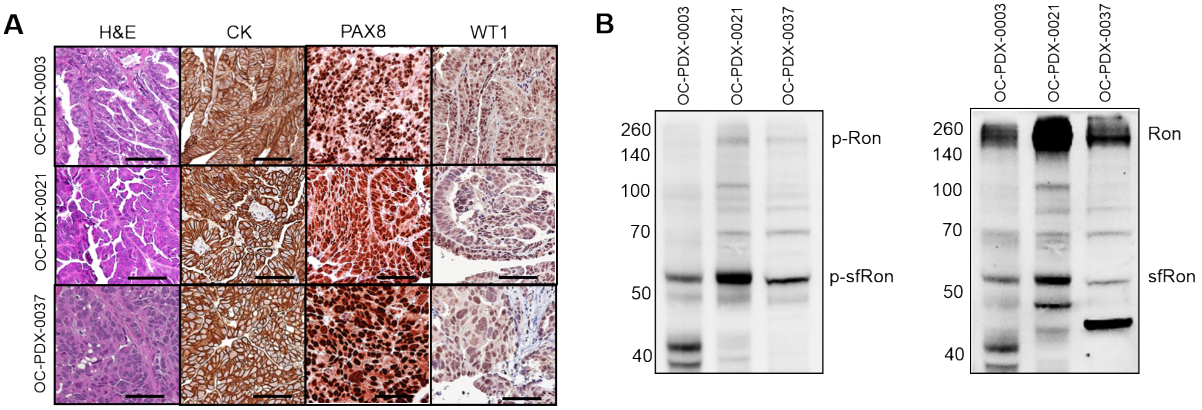

Supplementary Figure 3. Histological and immunohistochemical characterization of high-grade serous ovarian (HG-SOC) PDXs. A. The immunohistochemical analysis of the expression of commonly used markers for HG-SOC subtype such as pan-cytokeratin (CK), PAX8 and WT1 confirmed this specific tumor subtype. The images were taken at x 20 magnification and the scale bars represent 100 μm. **B.** Left panel displays western blot analysis of tumor lysates from the same HG-SOC PDXs as in A assayed for phosphorylated Ron isoforms. The blot was stripped and re-probed for total Ron (right panel).
